# Response of colonial Peruvian guano birds to flying UAVs: effects and feasibility for implementing new population monitoring methods

**DOI:** 10.7717/peerj.8129

**Published:** 2019-12-11

**Authors:** Cinthia Irigoin-Lovera, Diana M. Luna, Diego A. Acosta, Carlos B. Zavalaga

**Affiliations:** 1 Unidad de Investigación de Ecosistemas Marinos, Grupo de Aves Marinas, Universidad Cientifica del Sur, Lima, Lima, Peru; 2 Universidad Nacional Mayor de San Marcos, Lima, Lima, Peru

**Keywords:** Guano birds, Disturbance, Colonial birds, UAV, Aerial surveys, Peruvian islands, Cormorants, Boobies, Pelicans, Drone flights

## Abstract

**Background:**

Drones are reliable tools for estimating colonial seabird numbers. Although most research has focused on methods of improving the accuracy of bird counts, few studies have evaluated the impacts of these methods on bird behavior. In this study, we examined the effects of the DJI Phantom 3 drone approach (altitude, horizontal and vertical descent speeds) on changes in the intensity of behavioral response of guano birds: guanay cormorants (*Phalacrocorax bougainvilli*), Peruvian boobies (*Sula variegata*) and Peruvian pelicans (*Pelecanus thagus*). The breeding and non-breeding condition was also evaluated.

**Methods:**

Eleven locations along the Peruvian coast were visited in 2016–2017. Drone flight tests considered an altitude range from 5 to 80 m from the colony level, a horizontal speed range from 0.5 to 15 m/s, and a vertical descent speed range from 0.5 to 3 m/s. The intensity of the behavioral response of birds was scored and categorized as: 0-no reacting, 1-head pointing to the drone (HP), 2-wing flapping (WF), 3-walking/running (WR) and 4-taking-off/flying (TK). Drone noise at specific altitudes was recorded with a sound meter close to the colony to discriminate visual from auditory effects of the drone.

**Results:**

In 74% of all test flights (*N* = 507), guano birds did not react to the presence of the drone, whereas in the remaining flights, birds showed a sign of discomfort: HP (47.7%, *N* = 130), WF (18.5%), WR (16.9%) and TK (16.9%). For the drone approach tests, only flight altitude had a significant effect in the intensity of the behavioral response of guano birds (intensity behavioral response <2). No birds reacted at drone altitudes above 50 m from the colony. Birds, for all species either in breeding or non-breeding condition, reacted more often at altitudes of 5 and 10 m. Chick-rearing cormorants and pelicans were less sensitive than their non-breeding counterparts in the range of 5–30 m of drone altitude, but boobies reacted similarly irrespective of their condition. At 5 m above the colony, cormorants were more sensitive to the drone presence than the other two species. Horizontal and vertical flights at different speeds had negligible effects (intensity behavioral response <1). At 2 m above the ground, the noise of the cormorant colony was in average 71.34 ± 4.05 dB (*N* = 420). No significant differences were observed in the drone noise at different flight altitudes because the background noise of the colony was as loud as the drone.

**Conclusions:**

It is feasible to use the drone DJI Phantom 3 for surveys on the guano islands of Peru. We recommend performing drone flights at altitudes greater than 50 m from guano bird colonies and to select take-off spots far from gulls. Likewise, this study provides a first step to develop guidelines and protocols of drone use for other potential activities on the Peruvian guano islands and headlands such as surveys of other seabirds and pinnipeds, filming and surveillance.

## Introduction

Unmanned Aerial Vehicles (UAVs), or drones, are widely used for wildlife research (Koh & Wich, 2012; Chabot & Bird, 2015; Arts, Van Der Wal & Adams, 2015). High-resolution orthophotographic cameras and video equipment incorporated in UAVs have allowed for accurate estimates of the number and distribution of flora and fauna from a new perspective (Hodgson, Kelly & Peel, 2013; Evans et al., 2015; Chrétien, Théau & Ménard, 2016; Cruzan et al., 2016; Fiori et al., 2017; Stark et al., 2018). In seabirds, for instance, they have proven to be a reliable and a low-cost tool to estimate colony size with a minimum impact on the birds (McClelland et al., 2016; Hodgson et al., 2018). Perhaps one of the most advantageous features of UAVs for seabird biologists is that they can fly over remote colonies or locations of difficult access where birds breed (Ratcliffe et al., 2015; Brisson-Curadeu et al., 2017). These new findings help scientists and conservation managers to gain improved knowledge of the population size, distribution patterns and habitat use of seabirds (Muzaffar et al., 2017). Although most of the literature on UAV use for seabird research has focused on methods of improving the accuracy of bird counts (Chilvers et al., 2015; Goebel et al., 2015; Hodgson et al., 2018; Albores-Barajas et al., 2018) only a few studies have evaluated their impacts on bird behavior (Chabot, Craik & Bird, 2015; Rümmler et al., 2016; Borrelle & Fletcher, 2017; Barnas et al., 2018; Bevan et al., 2018). Overall, UAVs seem to have a negligible impact on seabirds or waterbirds; however, the behavioral response to UAV presence may vary according to the species surveyed (Weimerskirch, Prudor & Schull, 2018) annual-cycle condition (Brisson-Curadeu et al., 2017) UAV characteristics (McEvoy, Hall & McDonald, 2016) and UAV approaching conditions (speed, angle or altitude) (Vas et al., 2015).

In the cold nutrient-rich Humboldt Current System of Peru, there are 19–20 breeding species of seabirds (Crawford et al., 2006). The so-called guano birds, that is, the guanay cormorant (*Phalacrocorax bougainvillii*), the Peruvian booby (*Sula variegata*) and the Peruvian pelican (*Pelecanus thagus*), are among the most abundant. Their combined population once peaked at over 20 million birds in the 1950s (Duffy, 1994) although current estimates suggest that they do not exceed 5 million birds (Passuni et al., 2018). Cormorants, boobies and pelicans played an important role in the Peruvian economy for almost a century (1850s–1950s) due to the production of guano, a natural fertilizer harvested in almost all Peruvian islands and walled-off headlands (Duffy, 1994; Cushman, 2005). Given the commercial value of the guano birds, the government protects them since 1909 from any anthropogenic disturbance in their colonies (Coker, 1908; Ávila, 1954; Duffy, 1994). Estimates of guano bird numbers was of special interest for managers because of the close relation to guano production (Vogt, 1939, 1942). As a result, a complete sequence of guano bird numbers for the Peruvian coast have been collected since the early 1900s until now (Jordán & Fuentes, 1966; Tovar, Guillen & Nakama, 1987; Goya, 2000; Paleczny et al., 2015; Barbraud et al., 2018). Guano birds breed in large dense colonies of up to hundreds of thousands of pairs (Vogt, 1942) therefore, direct counts from the ground are useless for estimates of bird numbers. Rather, a planimetric method was implemented in the late 1950s (Jordán, 1963) and is still currently used. This method consists of sketching the shape of the bird colony in a scaled map. The area of the colony is then calculated and multiplied by the bird or nest density (Duffy, 1983; Tovar, Guillen & Nakama, 1987; Tovar & Cabrera, 2005). The observer is at the ground level and often has a limited view of the whole colony. To access a higher point for a better view, the observer may occasionally cause disturbance to the birds. Aerial photography of the guano bird colonies using kites or aircraft have been tried (Fraser et al., 1999; Delord et al., 2015) but are restricted by weather conditions or costs, respectively. With the arrival of UAV technology and high-definition aerial imagery in recent years, guano bird numbers may be more accurately estimated. We started testing drone flights over guano birds colonies in Peru in 2014 and have used them from 2016 to 2018 on different islands and headlands to evaluate count accuracy and disturbance.

In this study, we examine the effects of UAV approach on the intensity of behavioral response of Guanay cormorants, Peruvian boobies and Peruvian pelicans at 11 breeding sites along the Peruvian coast. UAV stationary altitude, horizontal speed and vertical descent speed were measured and related to the bird species and annual-cycle condition (breeders and non-breeders). Based on the observed human disturbance tolerance thresholds of cormorants, boobies and pelicans on the guano islands (Vogt, 1942) we hypothesized that species-specific behavioral responses to UAVs approach would be observed and differences would occur between breeding and non-breeding birds.

## Materials and Methods

This work was carried out under permits issued by Servicio Nacional de Áreas Naturales Protegidas por el Estado (SERNANP) through the Reserva Nacional Sistema de Islas, Islotes y Puntas Guaneras (RNSIIPG): R.J N°011-2016-SERNANP-RNSIIPG, R.J N°022-2016-SERNANP-RNSIIPG.

### Study site

**Figure 1:**
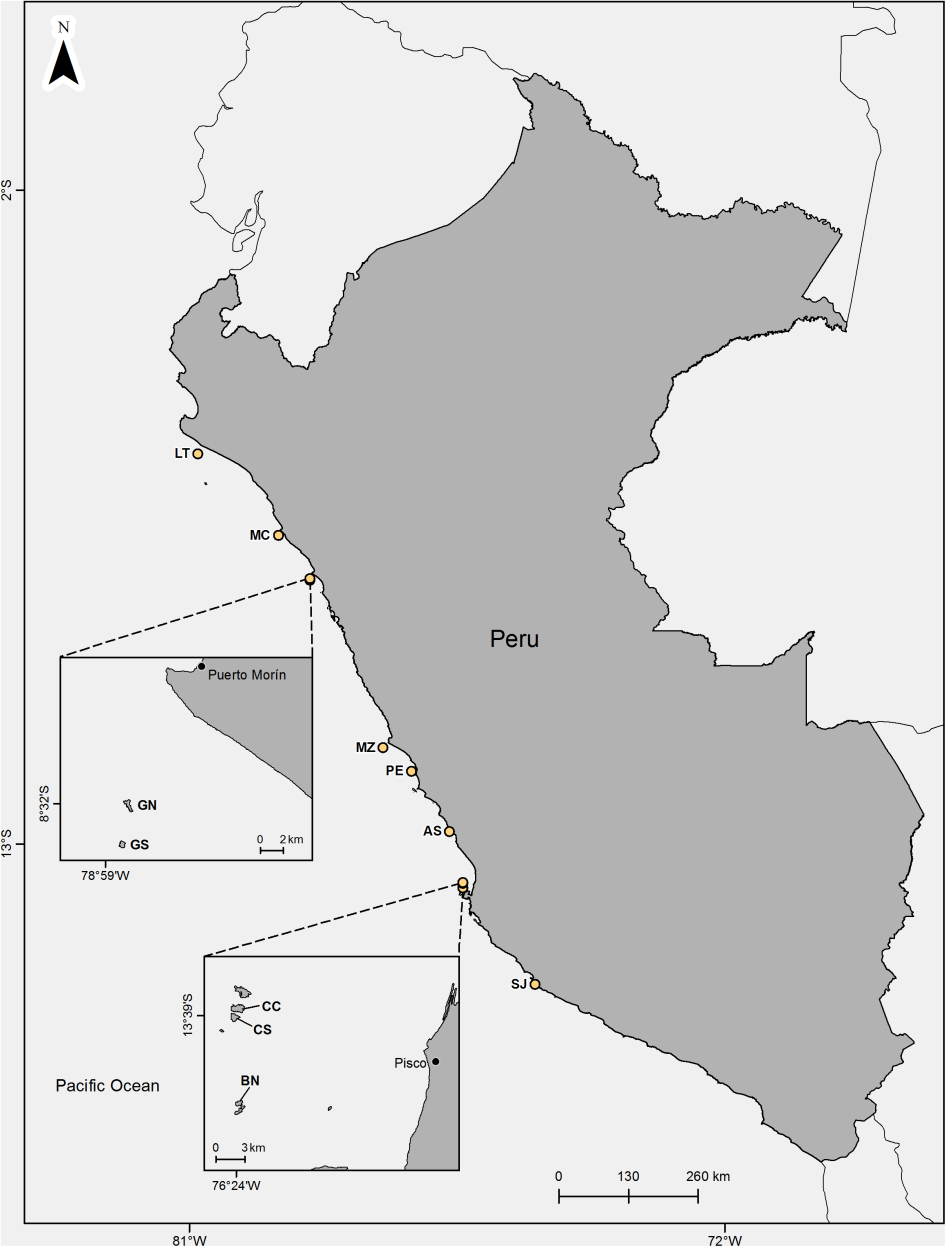
Location of the studied guano bird islands and headland off Perú. LT, Lobos de Tierra; MC, Macabi; GN, Guañape Norte; GS, Guañape Sur; MZ, Mazorca; PE, Pescadores; AS, Asia; CC, Chincha Centro; CS, Chincha Sur; BN, Ballestas Norte; SJ, P. San Juan. Map credit: Sebastián Lozano Sanllehi.

The study was carried out on 10 guano islands (Lobos de Tierra, Macabí, Guañape Norte, Guañape Sur, Mazorca, Pescadores, Asia, Chincha Centro, Chincha Sur y Ballestas Norte) and one guano walled-off headland (Punta San Juan) within the RNSIIPG (Fig. 1) between August 2016 and May 2017. The total number of visits was 17 for all sites: Isla Guañape Norte was visited three times, Isla Pescadores, Punta San Juan, Isla Mazorca and Isla Chincha Sur were visited twice, whereas the other six islands were visited once. Most of the studied islands are rocky but covered with a layer of guano produced mainly by the three species of guano birds. This guano is extracted from each place approximately every 5 years. The islands are barren, and many of them have vertical cliffs at 30–50 m high. The size of the visited islands ranges from 6.5 ha (Isla Macabí) to 1,570 ha (Isla Lobos de Tierra). In the interior of the islands, there are flat areas, slopes and hillsides. All colonies selected in this study were located on flat areas.

### Guano bird colony conditions

Guano birds are gregarious, forming large colonies up to hundreds of thousands of individuals. They can breed in mixed colonies, but in this study, we only selected mono-specific colonies for the tests to obtain independent behavioral responses for each species. Using the same criteria, we only chose colonies containing either breeding or non-breeding birds. The size of guano bird’s colonies in this study ranged from 2,000 to 140,000 individuals for cormorants, from 400 to 70,000 individuals for boobies and from 1,000 to 2,000 individuals for pelicans. The periphery of the colony (stripe from the edge to approximately 5 m inside the colony) was preferentially used for the drone tests as it allowed observers on the ground level a clear view of the birds at distances <100 m.

### Drone features

To study the effects of the drone on the birds, a white DJI Phantom 3 Professional quadcopter was used. The drone diagonal size was 35 cm and weight without battery was 1,280 g. It carried a Sony camera sensor EXMOR 1/2.3" (resolution: 12.4 MP, lens: f/2.8, 4K video, with 94° field of view) with stabilizer, barometer and a Globalnaya Navigazionnaya Sputnikovaya Sistema (GLONASS or Global Navigation Satellite System) navigation system (error <3 m). The drone was controlled manually by the pilot with a radio frequency (RF) remote control connected to a tablet, where the live transmission and flight parameters of the drone (distance, speed, altitude, etc.) were displayed. The camera always pointed downwards and perpendicular to the colony. Altitude measurements recorded by the drone were verified with a Bushnell rangefinder model 1 Arc Mile. The drone was elevated to different altitudes between 10 m and 50 m, and the altitude was recorded for both the drone and the rangefinder. The differences between both readings ranged from 10 cm to 90 cm (*r*^2^ = 0.99, *F*_1.5_ = 5423, *p* < 0.001, and = −0.44 + 1.0 ×) indicating that the altitude records of the drone were reliable for this study.

### Drone approach tests

A drone flight was defined as the total route from take-off to landing (modal flight time = 18 min). Multiple flights were performed at each visit and were mostly undertaken after dawn between 06:00 h and 11:00 h before birds left the colony for feeding. At this time of the day, we found the maximum number of birds in the colonies. The take-off point was usually located ~100 m from the colonies to prevent any reaction in the birds to our presence. A test is defined as the route and procedures taken by the drone within a flight to record the response of the birds to a given approach (flight altitude, horizontal speed and vertical descent speed). For example, one test may record the bird reaction at 10 m altitude or at a horizontal speed of 2 m/s. Each test was performed in different sectors of the colony, separated at least 50 m from each other to obtain independent records, so the birds were not preconditioned to the presence of the drone or influenced by previous tests. At this distance and based on our observations, it was unlikely to have birds from one sector reacting to the drone while being used in another sector.

**Figure 2:**
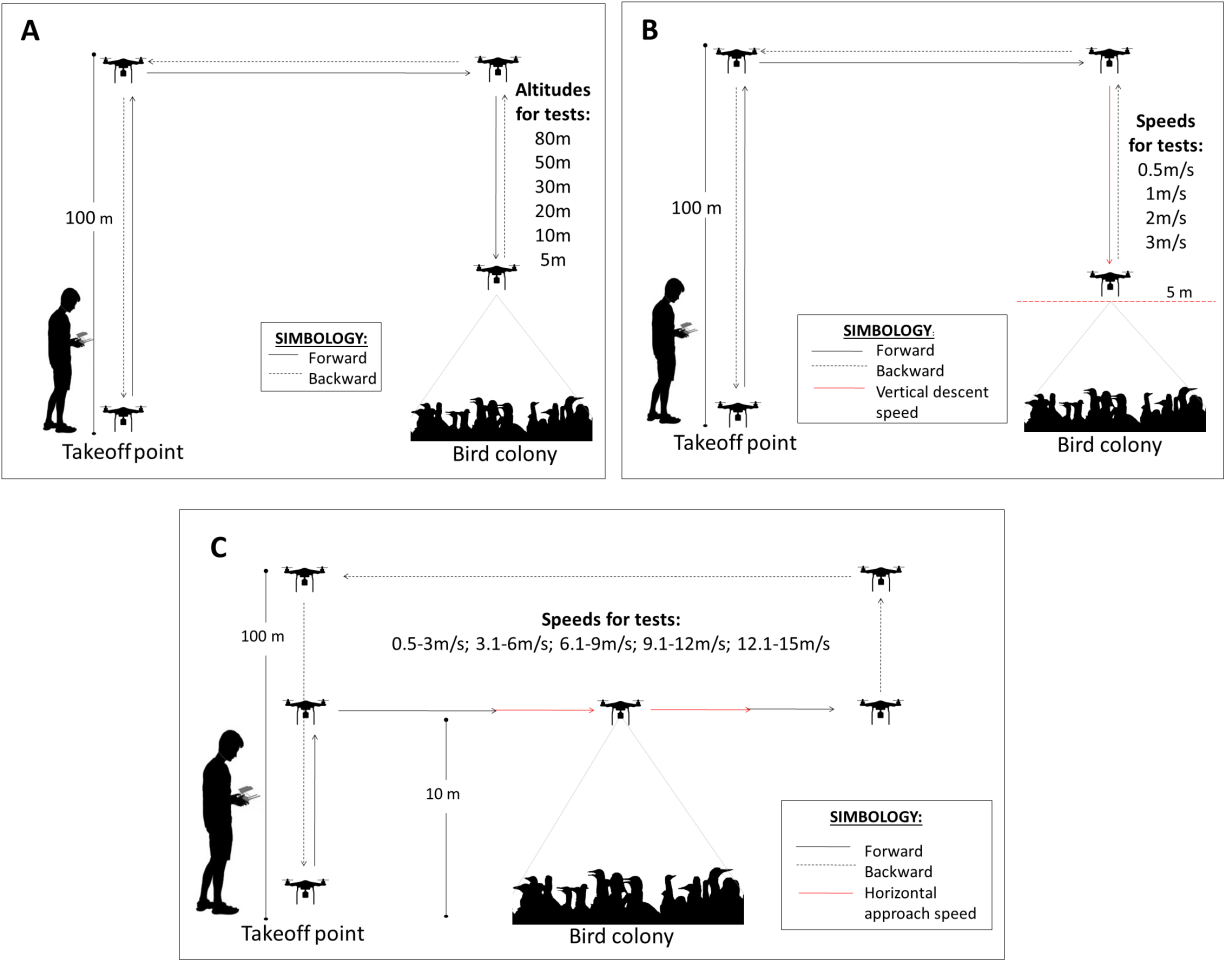
Drone flight protocols for the three drone approach tests. (A) Flight altitude (m), (B) vertical descent speed (m/s) and (C) horizontal approach speed (m/s).

For the flight altitude, the drone was located at 100 m above the level of the colony and descended at a speed of 0.5 m/s until the test altitude (80 m, 50 m, 30 m, 20 m, 10 m and 5 m). For each altitude, the drone remained stationary (hovering) for 1 min. Once the test was completed, the drone was lifted and moved to the take-off point to restart another test (Fig. 2A). For descent speed tests, we proceeded in the same way as for the altitude tests with the difference that the drone descended at different speeds (0.5 m/s, 1 m/s, 2 m/s and 3 m/s) up to 5 m over the colony (Fig. 2B). This value was chosen because birds begin to react more frequently at this altitude. For the horizontal speed tests, the drone was positioned first at a distance >50 m from the colony periphery and 10 m high from the colony. Then, the drone was accelerated until reaching a desired speed range and flew over the colony at 10 m above the birds. Because it was difficult to control the drone at a constant horizontal speed, ranges of speed were used for the tests: 0.5–3 m/s, 3.1–6 m/s, 6.1–9 m/s, 9.1–12 m/s and 12.1–15 m/s (Fig. 2C). The number of horizontal speed tests was less than the other tests because the horizontal displacement of the drone affected almost the entire colony and because in some locations, the presence of hillsides close to the colonies did not allow long horizontal flights.

### Bird responses to the drone approach

It was not possible to determine a proportion of the total number of birds in a colony sector that reacted to the drone approach because of the high density and large number of birds sampled in a particular test. Likewise, the number of birds enclosed within the tablet screen in a sampled colony sector varied with the drone altitude from ~1,800 birds at 80 m to <30 birds at 5 m, making accurate counts of birds and separation of disturbed and undisturbed birds challenging. Instead, the presence of a response was recorded when at least three birds within the studied sector of the colony showed any of these behaviors: no reaction (NR), head pointing to the drone (HP), wing flapping (WF), walking/running (WR) and taking-off/flying (TK). These categories were scored according to their intensity from 0 (NR) to 4 (TK). When birds displayed more than one response, the highest score was used for the analysis. This approach may have underestimate the scores at lower altitudes as finding 3 birds out of 1,800 with HP behavior may be more likely than finding 3 out of 30. Changes in bird behavior were observed in the tablet through live transmission from the drone. Simultaneous direct observations of the colony during the tests were made with the aid of 10 × 50 binoculars to confirm bird reactions.

### Explanatory variables

We examined whether the species of guano bird and status within the annual life cycle, that is, breeding and non-breeding adults could have an effect in the intensity of behavioral response. For the breeding condition, all tests were performed during the chick rearing phase. The three species overlapped in their breeding seasons, but also there was breeding asynchrony within and among species, making possible to find breeders and non-breeders on a same location in the majority of the visits.

### Effects of drone noise

To discriminate whether the effects of the drone on the response of the guanay cormorants were visual or auditory, sound tests were performed on isla Pescadores in five colonies containing breeding adults and chicks at different ages. First, the background noise of the colony was recorded in decibels (dB) during 5 min with a TENMARS TM-103 sound level meter. The sound meter was attached to a 10 m pole placed 2 m above and 5 m inside the colony periphery. Immediately afterwards, the drone used in the flight approaching tests was elevated over the sound meter spot in the cormorant colony at altitudes of 5 m, 10 m, 30 m, 50 m and 80 m, hovering for 1 min at each altitude to obtain sound records. Tests were repeated in the same colonies in two consecutive days.

### Statistical analysis

Drone approach tests were performed independently, so each data set (altitude, horizontal speed and vertical speed) had a different sample size and analyzed separately. Differences in the bird response intensity to the drone approach (from 0-no reaction to 4-taking-off/flying) were tested with a Generalized Mixed Model with a Restricted Maximum Likehood Estimation (REML) following a Poisson distribution due to the lack of heteroscedasticity of the data. Species, condition within the annual life cycle (breeding vs. non-breeding) and each of the drone approach type were set as fixed factors. Location was set as the random effect to cope with pseudoreplication as some islands were repeatedly visited. Likewise, a Generalized mixed model was used to test for differences in sound level of the drone across specific altitude categories (5 m, 10 m, 30 m, 50 m and 80 m). Colony ID was selected as random effect whereas flight category was defined as a fixed effect. The statistical package SAS 9.4 (SAS Institute, Cary, NC, USA) was used for all tests with the PROC GLIMMIX procedure to run mixed models. All tests were categorized as significant if *p* < 0.05.

## Results

A total of 507 flight tests were completed, of which 263, 152 and 92 were used to examine the effect of the flight altitude, vertical descent speed and horizontal approach speed, respectively. In 26% of the flights (*N* = 507), birds showed a sign of discomfort: HP (47.7%, *N* = 130), WF (18.5%), WR (16.9%) and TK (16.9%).

**Figure 3:**
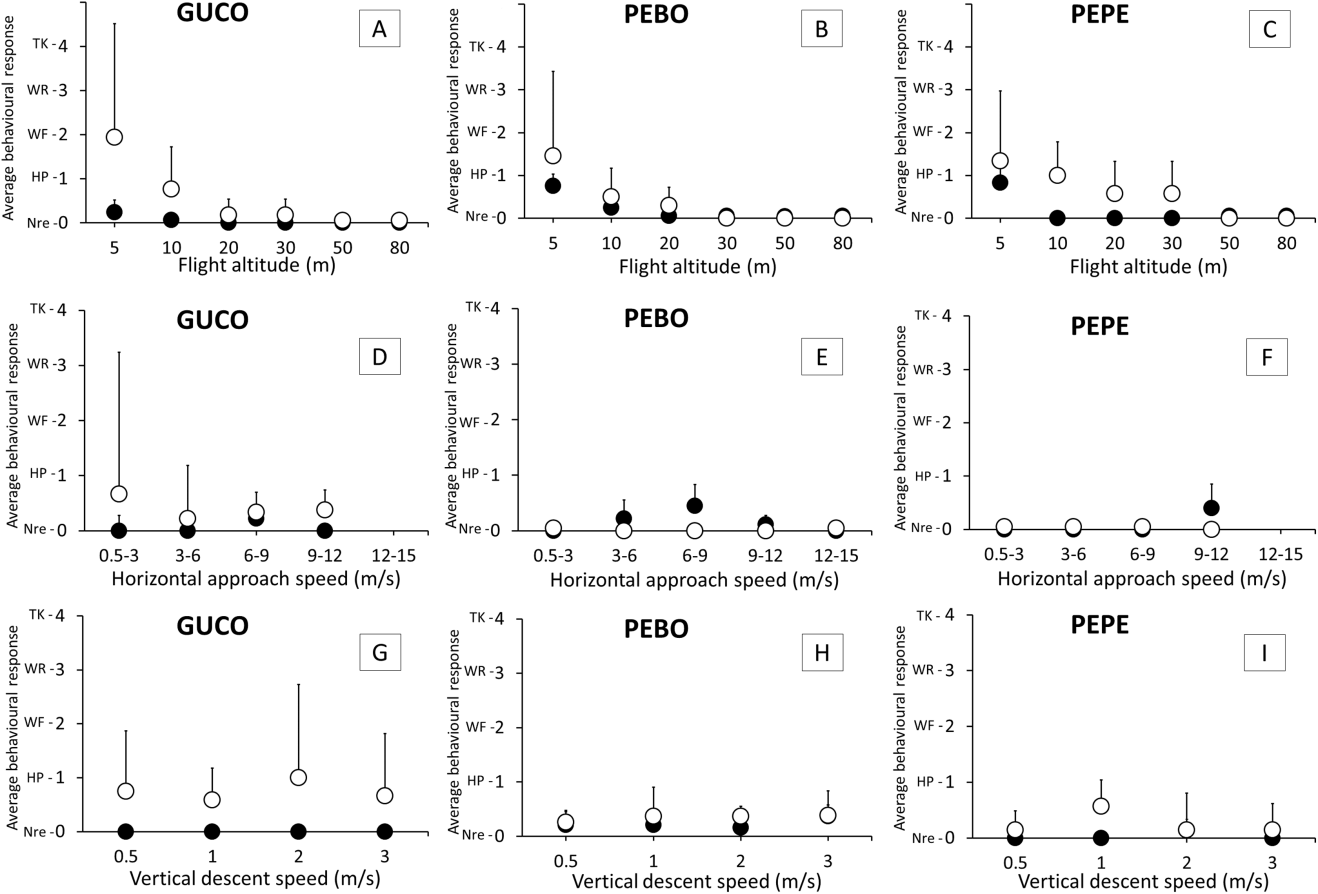
Intensity of the behavioral response of guano birds to drone approach: flight altitude, horizontal approach speed and vertical descent speed. GUCO, Guanay cormorant (A, D, G); PEBO, Peruvian booby (B, E, H); PEPE, Peruvian pelican (C, F, I). Black and white circles represent breeding (chick-rearing) and non-breeding birds. Behavioral response intensity was categorized as: Nre, no reaction; HP, head pointing to the drone; WF, wing flapping; WR, walking/running; TK, taking-off/flying. Values are average + 1 SE.

Overall, the average behavioral response intensity was low (<2 for all tests, Fig. 3) indicating that most of the birds either do not reacted, or raised their heads or flapped their wings before the presence of the drone.

### Flight altitude

Significant differences in the intensity of the behavioral response was observed for flight altitude (REML, *F*_5,217_ = 34.39, *p* < 0.001), species (REML, *F*_2,217_ = 3.23, *p* = 0.04), condition of the annual life cycle (REML, *F*_1,217_ = 25.29, *p* < 0.001), the interaction between flight altitude and condition (REML, *F*_5,217_ = 6.17, *p* < 0.001) and the interaction between species and condition (REML, *F*_1,217_ = 3.27, *p* = 0.04). No birds reacted at drone altitudes above 50 m from the colony. Birds, for all species and condition reacted more often at altitudes of 5 m and 10 m (Fig. 3). Chick-rearing cormorants and pelicans were less sensitive than their non-breeding counterparts in the range of 5–30 m (Fig. 3) but boobies reacted similarly irrespective of their condition. At 5 m above the colony, cormorants were more sensitive to the drone presence than the other two species (Fig. 3).

### Flight speeds

There were no significant effects of vertical descent speed (REML, *F*_3,119_ = 0.13, *p* = 0.95) nor horizontal speed (REML, *F*_4,58_ = 0.48, *p* = 0.75) in the intensity of behavioral response (<1, Fig. 3) that is, either birds did not react, or head pointed to the drone (Fig. 3).

### Drone noise

**Figure 4:**
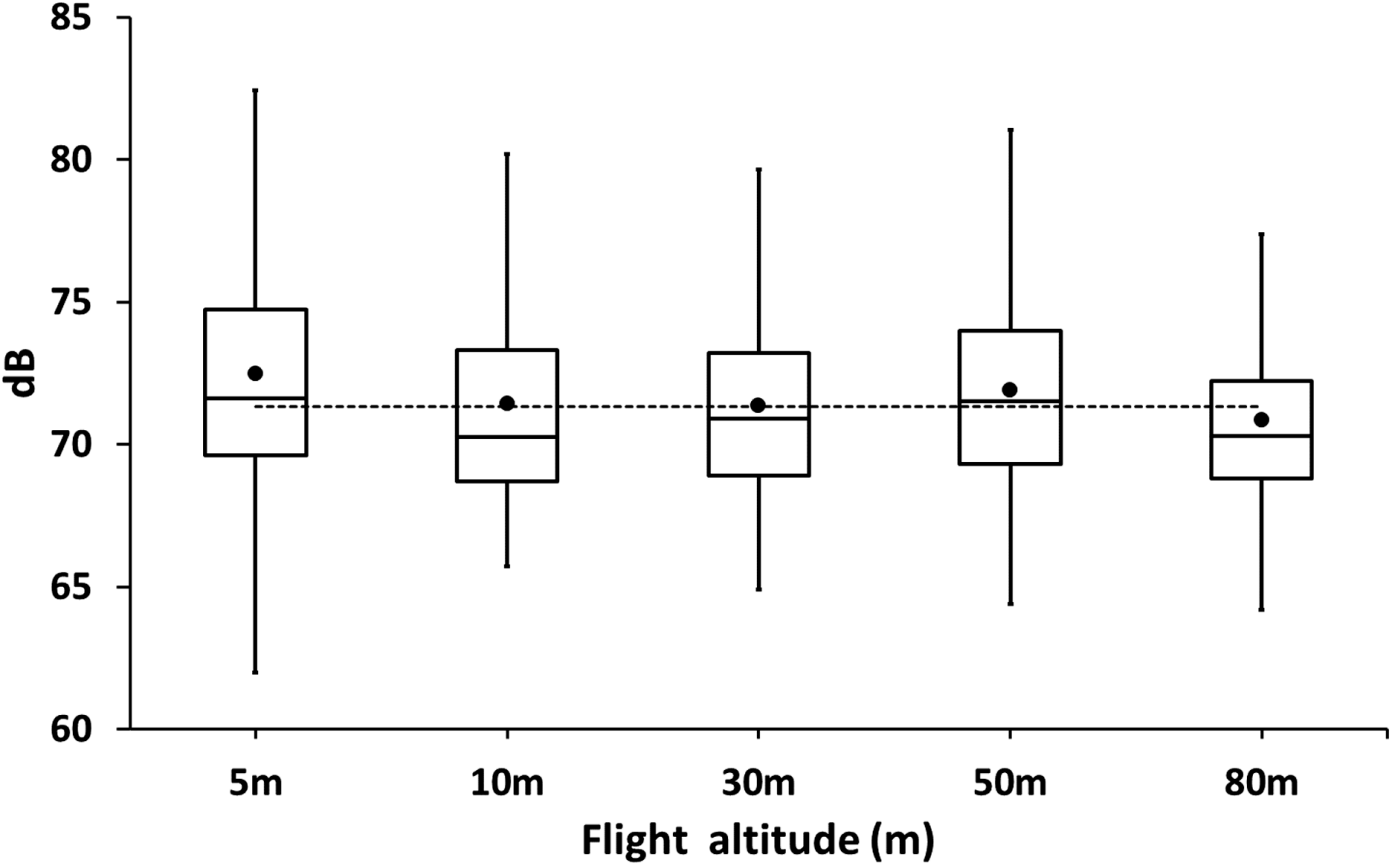
Box plot of sound levels (dB) measurements of drones hovering at different altitudes over a colony of breeding guanay cormorants with chicks on Isla Pescadores. The horizontal dotted line is the average background noise of the colony.

At 2 m above the ground, the noise of the cormorant colony was in average 71.34 ± 4.05 dB (*N* = 420). The colony was usually noisy as adults and chicks were always very active and vocalized loudly during the day (Fig. 4). No significant differences were observed in the drone at different flight altitudes (REML, *F*_5,24_ = 0.17, *p* = 0.97, Fig. 4) suggesting that the background noise of the colony was as loud as the drone even at close distance from the colony.

## Discussion

This study shows that, under some conditions, drones are feasible and reliable tools to perform guano bird counts on the Peruvian coast with a minimum degree of disturbance. Vertical and horizontal approaches up to 10 m above the colonies at different drone speeds did not have a significant impact on the birds’ behavior. On the other hand, the altitude tests revealed that the birds did not react to the presence of the drone at altitudes greater than 50 m above the colonies, and that the effects of the drone emerged at altitudes lower than 30 m. Given that the guano bird colonies are generally large (over thousands of individuals), drone flights higher than 50 m above the colonies would allow optimal photographs for counts without any harm to the birds. During our tests, several ortho photos were taken with the drone to estimate bird numbers and verified that in fact, birds were reliably counted at 50 m in altitude. Similar altitude thresholds or higher were also established in other seabird species such as penguins (Goebel et al., 2015; Borowicz et al., 2018) terns and frigate birds (Chabot, Craik & Bird, 2015; Hodgson et al., 2016).

The use of drones for population monitoring studies of seabirds began approximately 15 years ago, although related articles were only published a few years ago (Sardà-Palomera et al., 2012; Chabot & Bird, 2012; Goebel et al., 2015). In these works, emphasis is placed on the semi-automated quantification methods of birds in digital photographs of the colonies taken by the drones (Dulava, Bean & Richmond, 2015; Hurford, 2017). Recently, greater attention has been paid to the costs and benefits of using drones compared to other traditional count methods such as satellite image analysis, direct observation counts, transects and extrapolation (Chilvers et al., 2015; Hodgson et al., 2016) with greater emphasis on the disturbance drones may cause on birds (Lambertucci, Shepard & Wilson, 2015; Vas et al., 2015; McEvoy, Hall & McDonald, 2016; Brisson-Curadeu et al., 2017; Weimerskirch, Prudor & Schull, 2018; Bevan et al., 2018). The interest arises because many of the species under study have some type of conservation status and are found in natural protected areas; moreover, for ethical reasons, the temporary or permanent disturbance of these birds is to be avoided. The three species of guano birds are categorized as Endangered or Vulnerable in Perú Diario El Peruano (2014) DS N°004-2014-MINAGRI) and are prioritized species for conservation within the RNSIIPG (SERNANP, 2016). This study not only demonstrated that drones may be reliable tools for guano bird surveys because of their low impact in the colonies, but also provide an initial guideline for their use in other activities that can be potentially admitted in the RNSIIPG such as surveying other fauna, filming and surveillance.

The results of this study are similar to those found in other species and localities that evaluated the effects of flights of the DJI Phantom 3 Professional drone or similar models in various bird species. For example, in French lagoons, flamingos (*Phoenicopterus roseus*) and shorebirds (*Tringa nebularia*) in non-reproductive stages did not react to the approach of drones at altitudes greater than 50 m, although the birds were affected by the approach angle (Vas et al., 2015). On Natividad Island in Mexico, western gulls (*Larus occidentalis*) also completely ignored the presence of drones at elevations greater than 60 m (Albores-Barajas et al., 2018). Moreover, drone flights higher than 50 m did not cause a reaction in several species of Anseriformes in Australia (McEvoy, Hall & McDonald, 2016). In other studies, conducted in Australia with terns (*Thalaseus bergii*), the birds were only disturbed at altitudes of less than 60 m (Bevan et al., 2018). Finally, in the sub-Antarctic Possession Island, imperial cormorants (*Phalacrocorax atriceps*) were alerted to the drones at altitudes less than 25 m (Weimerskirch, Prudor & Schull, 2018). Signs of behavioral stress were more frequently observed in non-breeding adults than in breeders during drone approach trials (Weimerskirch, Prudor & Schull, 2018; Brisson-Curadeu et al., 2017) a pattern also observed in our study. These differences in behavior were expected in guano birds as breeding birds are more tolerant to disturbance than their non-breeding counterparts, which usually flush and fly-off to human presence (Vogt, 1939). Likewise, it is interesting to note that adult guano birds are not vulnerable to predation that cause a greater state of alertness, which may explain their high tolerance to drone flights. In the past, Andean condors (*Vultur gryphus*) visited the guano islands and provoked the desertion of cormorant colonies (Murphy, 1931) however, this effect does not longer occur because of the low number of condors in the Peruvian coast relative to past decades.

The results obtained in this study are restricted to the use of the DJI Phantom 3 Professional drone or to quadcopter drones of similar characteristics. Drones of different shapes, sizes, colors and sound emission may cause different reactions in guano birds. In species of ducks and swans in lagoons of Australia, the most important characteristics that provoked a reaction was the shape and size of the drone more than the color or sound (McEvoy, Hall & McDonald, 2016). Indirect effects of drone flights on guano birds were also observed when it took-off near territorial seabird species. In some trials, drone flights far from the colonies of guano birds triggered alarm calls of Peruvian gulls (*Larus belcheri*), particularly during their breeding season (october–february). Occasionally, gulls chased the drone up to the guano bird colonies and their calls, flushed out the cormorants and boobies.

It is important to remark that this study only focused on behavioral observations. Guano birds with no signs of stress during the drone approach tests may hide unobserved physiological responses. King penguins (*Aptenodytes patagonicus*) showed a significant increase in heart rates during drone approach tests, despite the lack of behavioral responses (Weimerskirch, Prudor & Schull, 2018). Further studies addressing the correlation between behavioral and physiological responses on guano birds during drone approach flights are recommended to adjust on the use of drones in the guano bird colonies.

## Conclusions

The effects of UAV approach on the behavioral response of guanay cormorants, Peruvian boobies and Peruvian pelicans were evaluated at 11 breeding sites along the Peruvian coast. Significant signs of discomfort were recorded only at drone flight altitudes <30 m and the behavioral responses could vary according to the species and their annual-cycle condition. No changes in bird behavior were observed at flights >50 m. Other variables such as horizontal speed and vertical descent speed did not lead to changes in the intensity of behavioral responses to the drone. Any sign of bird discomfort was attributed to visual rather than auditory contact with the drone.

These findings demonstrate that it is feasible to use the drone DJI Phantom 3 Professional for bird surveys on the guano islands of Peru. Based on these results, we recommend performing drone flights at altitudes greater than 50 m from guano bird colonies and to select take-off spots far from Peruvian gulls. Likewise, this study provides a first step to develop guidelines and protocols of drone use within the RNSIIPG for other potential activities such as surveys of other seabirds and pinnipeds, filming and surveillance.

## Supplemental Information

10.7717/peerj.8129/supp-1 Supplemental Information 1 Data collected in the field.

## Additional Information and Declarations

### Field Study Permissions

The following information was supplied relating to field study approvals (i.e., approving body and any reference numbers): Field experiments were approved by the Servicio Nacional de Áreas Protegidas por el Estado through the Reserva Nacional Sistema de Islas, Islotes y Puntas guaneras (Chief Resolution N°011-2016-SERNANP-RNSIIPG and Chief Resolution N°022-2016-SERNANP-RNSIIPG).

### Data Availability

The following information was supplied regarding data availability: The raw measurements are available in the Supplemental File.
